# Non-contrast 3D T1-weighted MRI at 3T for early detection of atrial lesions post-ablation in atrial fibrillation: a pilot study

**DOI:** 10.1007/s10554-026-03625-9

**Published:** 2026-01-27

**Authors:** Jean-François Deux, Miklos Kassai, Nicolas Johner, Carl Glessgen, Lindsey Alexandra Crowe, Karl-Philipp Kunze, René Michael Botnar, Claudia Prieto, Dipen Shah, Jean-Paul Vallée

**Affiliations:** 1 Department of Radiology, Geneva University Hospitals, Rue Gabrielle-Perret-Gentil 4, 1205 Geneva, Switzerland; 2 Department of Cardiology, Geneva University Hospitals, Rue Gabrielle-Perret-Gentil 4, 1205 Geneva, Switzerland; 3 MR Research Collaborations, Siemens Healthcare Limited, Camberley, UK; 4 School of Biomedical Engineering and Imaging Sciences, King’s College London, London, UK; 5 Millenium Institute for Intelligent Healthcare Engineering, Santiago, Chile; 6 Institute for Biological and Medical Engineering, Pontificia Universidad Católica de Chile, Santiago, Chile

**Keywords:** Atrial fibrillation, Magnetic resonance imaging/methods, Heart/diagnostic imaging, Catheter ablation, Contrast media/adverse effects

## Abstract

To assess the feasibility of a native 3D T1-weighted Bright-blood and black-blOOd phase SensiTive (BOOST) cardiac MRI sequence for detecting acute atrial lesions immediately after radiofrequency ablation (RFA), without gadolinium contrast administration. Ten atrial fibrillation patients underwent 3T cardiac MRI within one hour following RFA. Whole-heart black-blood T1-weighted BOOST and late gadolinium enhanced (LGE) images were acquired during free breathing. Two experienced readers independently assessed the presence and continuity of acute lesions in six predefined pulmonary vein (PV) antrum quadrants per patient. Findings were correlated with procedural RFA parameters. BOOST and LGE sequences detected high signal intensity lesions in all patients, involving both right and left PV antra. BOOST identified acute lesions in 62 and 63% of right and left PV quadrants, respectively, against 100% of quadrants with LGE. Over 80% of LGE-positive quadrants also showed microvascular obstruction. Quadrants with absent or discontinuous BOOST lesions were associated with a significantly greater inter-lesion distance and lower impedance drop during RFA compared to quadrants with continuous lesions. Whole-heart black-blood T1-weighted BOOST cardiac MRI at 3T enables detection of acute PV antrum lesions immediately after RFA without contrast administration. This contrast-free imaging technique may enable real-time, gadolinium-free lesion assessment during or immediately after RFA.

**Graphical abstract:**

**Figure:**
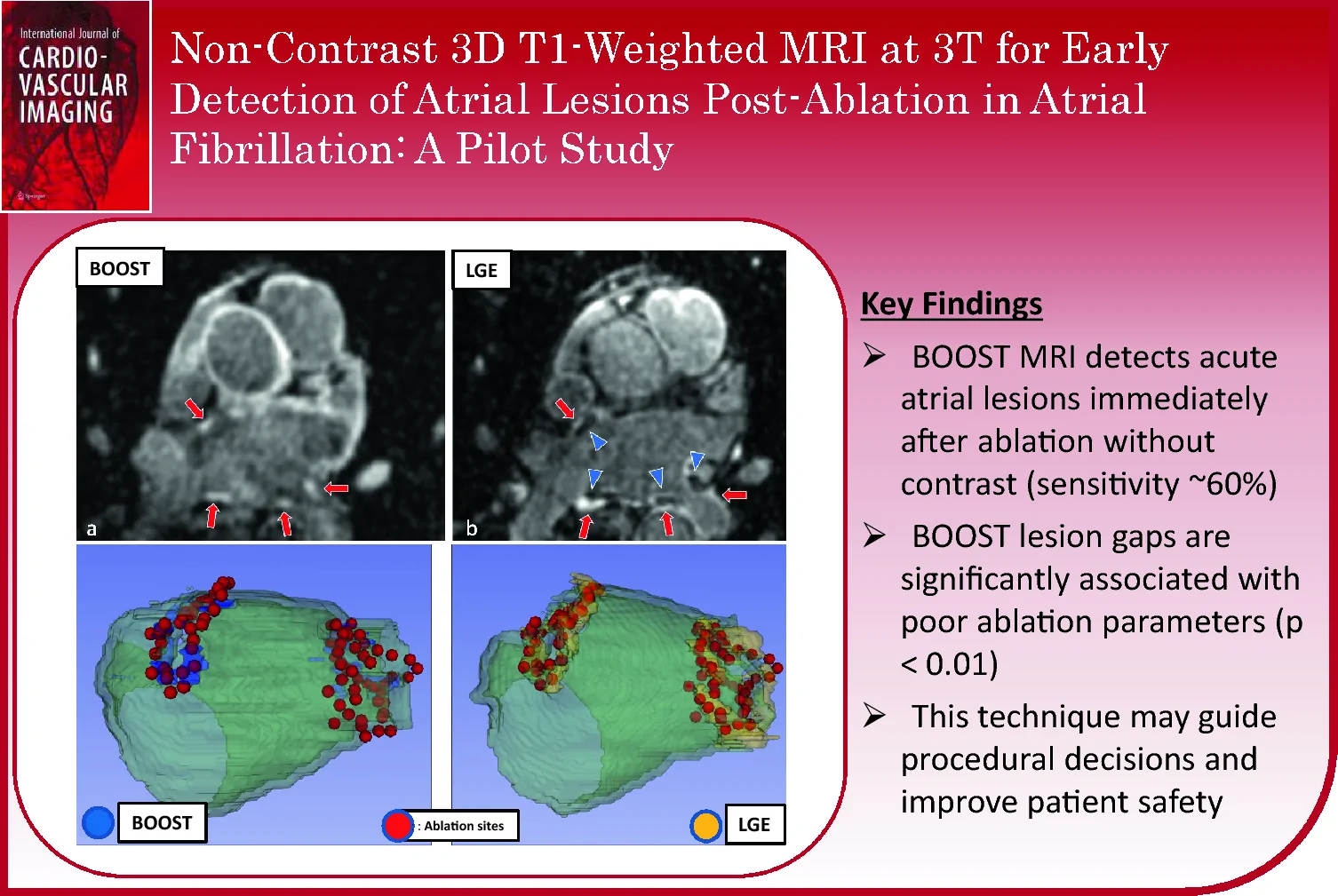

## Introduction

Radiofrequency catheter ablation (RFA) is an effective therapeutic option for selected patients with atrial fibrillation (AF) [1, 2], yet a significant proportion of these patients experience recurrence of AF following the procedure [3, 4]. Such recurrences are frequently attributed to pulmonary vein (PV) reconnection [5, 6], resulting from residual gaps of viable myocardium within ablation-induced scars. Cardiac MRI performed several months after RFA (> 3 months) can identify scar tissue in the pulmonary vein walls, which as areas of high signal intensity on T1-weighted images after contrast injection [7–9]. Several studies have also reported that MRI may provide prognostic information on post-ablation recurrence, related to the amount of pre-ablation fibrosis [10], the presence of circumferential scarring around the veins [11], and the extent of residual fibrosis without scarring after ablation [12]. Moreover, early cardiac MRI performed immediately after or during the RFA procedure can detect acute atrial lesions using gadolinium-enhanced sequences [13–16]. Identifying incomplete lesion formation at this early stage can guide clinical decision-making, such as prompting additional targeted ablation to reduce the risk of recurrence [17, 18]. Hence, cardiac MRI plays a crucial role in both immediate assessment and long-term follow-up, helping to optimize therapeutic outcomes for patients undergoing AF ablation.

Although late gadolinium enhancement (LGE) is currently the gold standard for visualizing acute lesions, its clinical utility is constrained by contrast-agent-related safety concerns [19], the need for repeated gadolinium injections to recheck new lesions or procedural gaps, and the issue of microvascular obstruction (MVO), which can hinder the detection of recent lesions. Gadolinium administration could be avoided by using a 3D long inversion recovery time (TI) T1-weighted MR sequence without injection, exploiting the T1 effect of hemorrhagic necrosis at 1.5T. While promising results have been demonstrated in animal models following RFA [20–22], human data remain scarce, with only one clinical study reporting acute T1-weighted atrial lesions post-RFA at 1.5T [23].

The 3D Bright-blood and black-blOOd phase SensiTive (BOOST) research sequence is a GRE-Dixon-based technique that simultaneously generates bright-blood anatomical images and dark-blood T1-weighted images [24]. BOOST bright-blood imaging has shown encouraging results for detecting atrial thrombi and assessing pulmonary venous anatomy prior to RFA [25]. However, the clinical utility of BOOST dark-blood T1-weighted images remains underexplored.

In this study, we hypothesized that BOOST dark-blood T1-weighted imaging could identify acute atrial lesions immediately after RFA, without requiring gadolinium injection, by exploiting the T1 effects of hemorrhagic necrosis. We conducted a prospective study using a 3T MRI system, which allows faster acquisitions and potentially improved detection of acute lesions without contrast administration.

## Methods

This prospective study was approved by our local ethics committee (registration number: 2017-01177; 9/10/2017), and full written informed consent was obtained from each patient.

### Patient population

Between April 2022 and September 2023, 11 consecutive cases of patients undergoing AF ablation with periprocedural cardiac MRI were included; All patients were examined immediately (< 60 min) after RFA with cardiac MRI on a 3T Intraoperative magnetic resonance imaging system (iMRIS) system located next to the electrophysiology laboratory.

### Ablation procedure

Catheter ablation was performed using the institutional protocol [26]. Transfemoral, transseptal access was obtained under fluoroscopy. A 10-electrode catheter was positioned in the coronary sinus, and another in the low right atrium. A 3D electroanatomical map (CARTO, Biosense Webster) of the left atrium and PVs was acquired using a circular LASSO catheter. Circumferential antral PV isolation was performed with a SmartTouch SF catheter (25–35 W, 20–30 s, target impedance drop: 10 Ω, interlesion distance ≤ 4 mm). Post-procedural maps were reviewed to assess maximum interlesion distance (mm), minimum impedance drop (Ohm), Ablation Index (dimensionless), and Force–Time Integral per quadrant (g*s). RF tags < 10 s were excluded.

### MR imaging

Cardiac MRI was performed under general anesthesia using a ceiling-mounted IMRIS 3T system (MAGNETOM Skyra, Siemens Healthineers, Erlangen, Germany) with a high-performance gradient (40 mT/m; slew rate 200 mT/m/s) and a 6-channel cardiac coil. Patients were transferred on the MRI-compatible catheter table directly into the MRI suite after safety clearance by a technician using a dedicated checklist.

The BOOST research sequence was acquired on the whole heart in the coronal plane with ECG gating and in free breathing using image-based navigation (iNAV) to enable non-rigid respiratory motion correction and 100% respiratory scan efficiency. The coronal orientation was chosen because the BOOST acquisition had to be aligned with the respiratory motion navigator, which operates exclusively in the coronal plane over the left ventricle to track cardiac displacement during respiration. This sequence generates two differently weighted bright-blood volumes acquired from two successive heartbeats in an interleaved fashion: a first volume with T2 prep-IR preparation a second without preparation. Both are then combined using a magnitude subtraction-based approach (iT2prep) to provide a third, 3D T1-weighted black-blood volume [24, 25]. A bipolar GRE-Dixon readout is used at in- and opposed phase echo times, and Dixon-based fat–water separation is performed after non-rigid motion-compensated reconstruction of each echo based on the iNAV motion information. Following BOOST, the same GRE-Dixon-based acquisition and reconstruction approach was used for a 3D Late Gadolinium Enhanced (LGE) Inversion Recovery (IR) sequence, acquired in axial orientation 15 min after gadolinium administration (Dotarem®, Gadoteric acid; Guerbet, France; 0.2 mmol/kg). A dedicated TI scouting sequence was systematically performed to adjust the optimal TI. The parameters of BOOST and LGE sequences are reported in Table 1.

**Table 1:** Sequence parameters of BOOST and LGE sequences

	BOOST sequence	LGE sequence
TR (ms)	4.47	5.04
TE (ms)	1.3/2.8	1.3/2.8
Fat saturation	Dixon	Dixon
BW (Hz/Px)	698	849/814
Nb of slices	72	104 ± 12
Matrix	224 * 224	256 * 256
Pixel size (mm)	1.4 × 1.4 × 1.4	1.3 × 1.3 × 1.3
Duration (min)	8 min 14 s ± 56 s	7 min 52 s ± 1 min
TI	140	288 ± 33
Orientation	Coronal	Axial
Navigator	iNav	iNav

The reported spatial resolutions correspond to the true voxel dimensions during acquisition, without interpolation, zero-filling, or partial slice reconstruction

*BW* bandwidth, *TE* echo time, *TI* inversion recovery time, *TR* repetition time

### Qualitative image analysis

Whole-heart dark-blood T1-weighted BOOST and LGE images were anonymized and blindly analyzed in consensus by two cardiac experts (JFD; CG) with 10 and 5 years of experience in cardiac MRI, respectively, using a dedicated software (OsirX, Pixmeo).

#### Image quality

BOOST and LGE image quality was graded on a 4-point scale: 1 = poor (non-diagnostic), 2 = fair (readable despite artifacts), 3 = good (minor artifacts), 4 = excellent (no artifacts).

#### PV antrum quadrant analysis

PV antra were reconstructed using multiplanar reformatting using OsiriX (Pixmeo, Switzerland) and divided into six quadrants (superior, antero-superior, antero-inferior, inferior, postero-inferior, postero-superior). Each was assessed for presence and continuity of acute lesions, defined as high signal intensity with wall thickening (compared to the anterior atrial wall). For LGE, low signal areas suggesting MVO were also evaluated. Image analysis was repeated twice by two readers 6 months apart to assess reproducibility.

### Quantitative image analysis

**Fig. 1:**
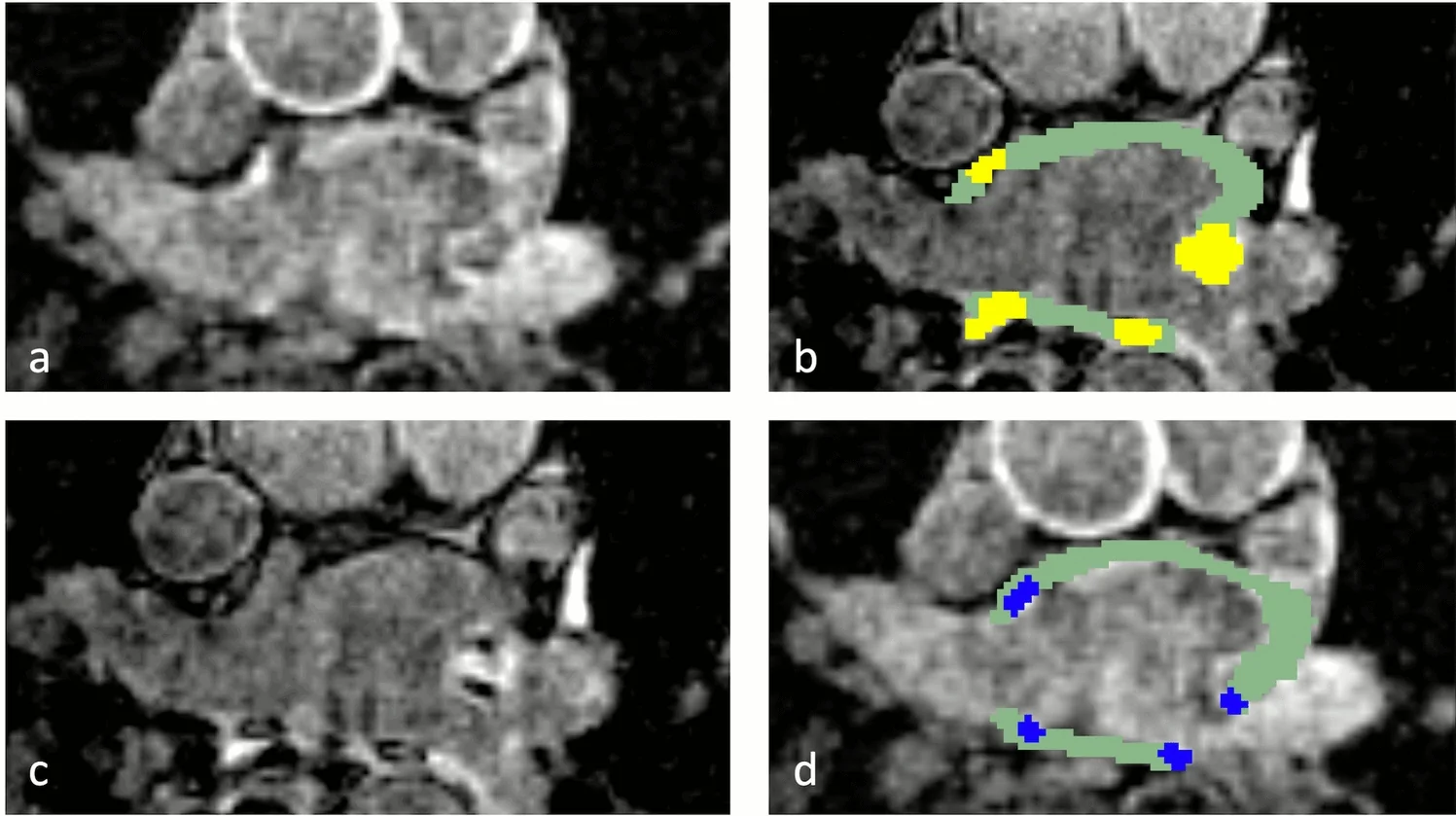
Example of manual lesion segmentation on BOOST and LGE images in a representative patient. Top row: BOOST images before (left) and after (right) manual segmentation. Hyperintense atrial wall regions corresponding to acute lesions were manually delineated slice by slice (blue areas) on multiplanar reformats, while the antral wall surface is shown in green for orientation. Bottom row: Corresponding LGE images before (left) and after (right) manual segmentation (yellow areas). The segmented regions were used to calculate lesion volumes and to generate three-dimensional reconstructions of the atrial wall

Signal-to-noise ratio (SNR) was defined as the mean signal intensity (SI) of acute lesions divided by background noise, measured as the standard deviation of SI in air anterior to the chest wall. Signal-to-blood ratio (SBR) and two contrast-to-noise ratios (CNR) for lesions vs. blood and myocardium were calculated. Lesion thickness was measured on BOOST and LGE images by the same two readers. Total lesion volume was assessed by manual delineation of high–signal–intensity regions on multiplanar reformats using 3D Slicer (version 5.6.2; www.slicer.org) [27] (Fig. 1). When present within LGE lesions, MVO areas were included in the overall segmentation, and their individual volumes were also measured separately. In addition, atrial walls were manually segmented to enable 3D reconstruction of the left atrium and fusion with the electroanatomical map.

### Statistical analysis

Data are reported as mean ± standard deviation or median (interquartile range), depending on the data distribution, or percentages. The normality of the data was assessed using the Shapiro–Wilk test. Paired t-tests were used to compare minimum impedance drop during ablation procedure, lesion thickness, total volume measurements, SNR and CNR ratios. The non-parametric Wilcoxon test was used for other groups comparison. Pearson correlation coefficients were calculated to assess the correlation between continuous variables. Inter-rater agreement for the presence of acute lesions on BOOST images was assessed using weighted (squared) Cohen’s Kappa. Intraclass correlation coefficients (ICCs) were used to determine absolute inter-reader agreement for the volume of BOOST and LGE lesions. BOOST and LGE lesions were categorized as continuous or discontinuous using a quadrant-based analysis, and the corresponding electrophysiological parameters (maximum inter-lesion distance, minimum impedance drop, Ablation Index, and Force–Time Integral per quadrant) were compared between groups. A P-value < 0.05 was used for statistical significance. SPSS software version 20.0, (SPSS, Chicago, Ill, USA) was used for all analyses.

## Results

A total of 10 patients were included (mean age: 63.8 ± 8.9 years, 90% male). One patient was excluded due to respiratory artifacts that precluded image analysis on both sequences. Baseline clinical and procedural characteristics are summarized in Table 2.

**Table 2:** Patients characteristics (N = 10)

Age (years)	63.8 ± 8.9
Male sex	9 (90%)
Body mass index (kg/m^2^)	27.7 ± 4.9
Paroxysmal AF	10 (100%)
Months since first diagnosis of AF	27 (13.3–53)
CHA_2_DS_2_-VASc score	1.5 (0–3)
Number of failed AADs	2 (2–2)
Comorbidity	
Arterial hypertension	4 (40%)
Diabetes mellitus	1 (10%)
Prior stroke/TIA	1 (10%)
Structural heart disease*	2 (20%)
Transthoracic echocardiography	
Left ventricular ejection fraction (%)	60.0 ± 3.5
Left atrial volume index (ml/m^2^)	33.8 ± 9.7
Procedural data	
Procedure duration (min)	200 (157.5–240)
Radiofrequency time (min)	51.6 ± 13.3
PVI achieved	10 (100%)
Cavotricuspid isthmus ablation	7 (70%)
Substrate modification**	5 (50%)

*AAD* antiarrhythmic drug, *AF* atrial fibrillation, *CHA*_*2*_*DS*_*2*_*-VASc* congestive heart failure, age ≥ 65 years, diabetes, history of stroke, vascular disease, age ≥ 75 years, female gender, *PVI* pulmonary vein isolation, *TIA* transient ischemic attack

Data are mean ± SD. median (IQR) or n (%)

*Defined as any of the following: coronary artery disease, cardiomyopathy, hemodynamically significant valvular heart disease, heart failure

**Includes fractionated electrogram ablation in 5 patients, of which 2 also underwent posterior wall linear lesion (connecting PVI lesions) and 1 lateral right atrial linear lesion (connecting to the inferior vena cava)

### Qualitative lesion detection

**Fig. 2:**
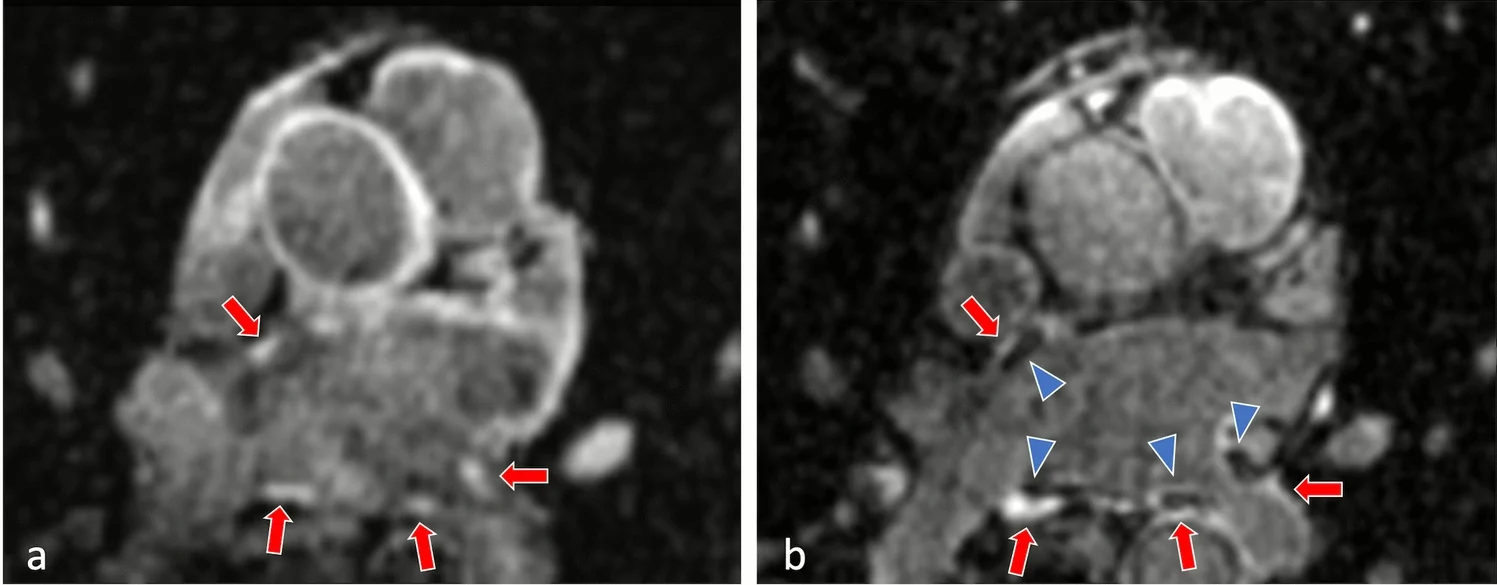
Example of high-intensity acute atrial lesions (arrows) detected around the PVs on BOOST (**a**) and LGE (**b**) MR images acquired immediately after radiofrequency ablation in a 78-year-old patient. Low SI areas suggestive of MVO lesions were also detected within the LGE lesions (**b**, arrowheads)

**Fig. 3:**
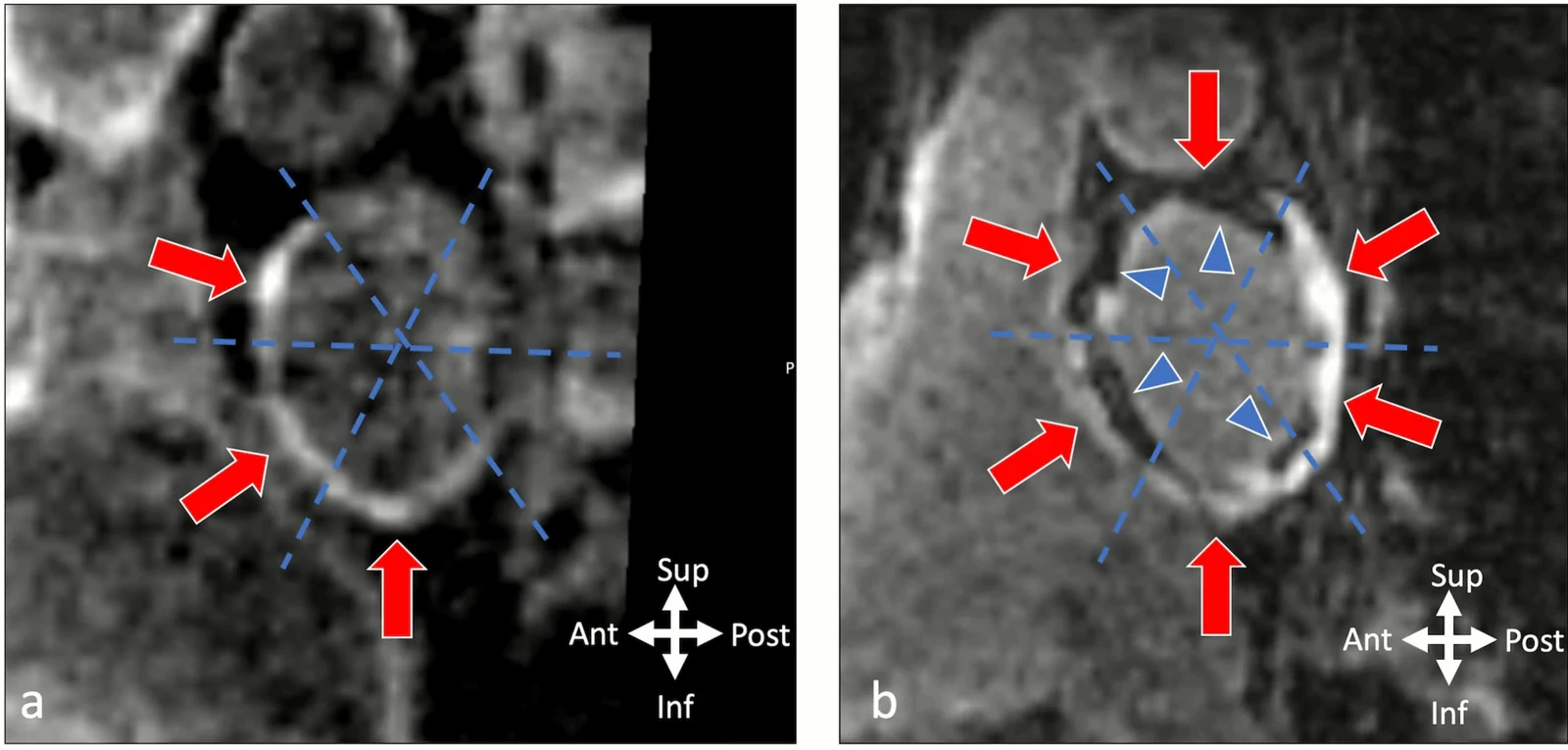
Example of early atrial lesions detected around the antrum of both right PVs on BOOST (**a**) and LGE (**b**) MR images acquired immediately after radiofrequency ablation in a 70-year-old patient. Three quadrants (superior, antero-superior, antero-inferior) were considered positive (i.e., containing an acute lesion) on BOOST images (**a**, arrows). All 6 quadrants of the right PV antrum were considered positive on LGE image (**b**, arrows). Note the presence of low signal intensity components within high signal intensity lesions on the LGE image, suggestive of MVO lesions (arrowheads). Dashed lines delineate the six pulmonary vein antrum quadrants

**Fig. 4:**
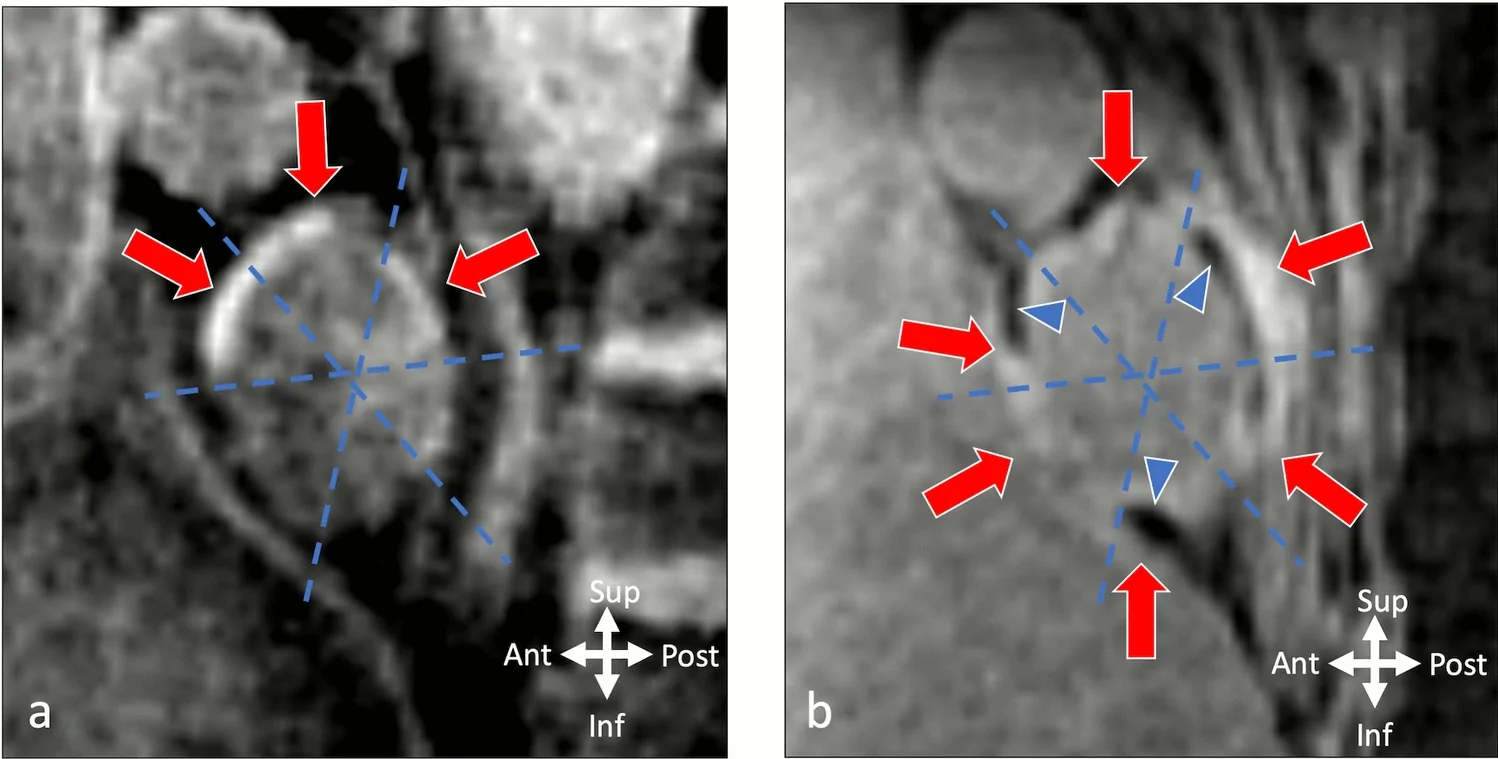
Example of early atrial lesions detected around the antrum of both right PVs on BOOST (**a**) and LGE (**b**) MR images acquired immediately after radiofrequency ablation in a 63-year-old patient. Three quadrants (superior, antero-superior, postero-superior) were considered positive on BOOST images (**a**, arrows). All 6 quadrants of the right PV antrum were considered positive on LGE image (**b**, arrows). MVO lesions (arrowheads) were present within high-intensity LGE lesions. Dashed lines delineate the six pulmonary vein antrum quadrants

There was no significant difference in overall image quality between BOOST and LGE (3.1 ± 0.3 vs. 3.4 ± 0.9; P = 0.2). Acute atrial lesions were consistently visualized in the pulmonary vein (PV) antra on both BOOST and LGE images in all patients (Fig. 2). On BOOST, 62% (37/60) of right PV antral quadrants and 63% (38/60) of left quadrants were classified as positive for acute lesions. The highest positivity rates were observed in the antero-superior quadrants (100% on the right, 90% on the left), while the lowest were seen in the postero-inferior quadrants (40 and 30%, respectively). A continuous lesion pattern was identified in 45% (27/60) of right-sided and 30% (18/60) of left-sided quadrants (Figs. 3 and 4). In contrast, LGE imaging revealed high signal intensity lesions in all 60 quadrants of both PV antra. These lesions appeared continuous in all cases and were frequently associated with microvascular obstruction (MVO), detected in 87% (52/60) of right-sided and 82% (49/60) of left-sided quadrants. Among MVO-positive quadrants, 70% (42/60) on the right and 67% (40/60) on the left exhibited a continuous pattern. Detailed quadrant-level findings are provided in Table 3.

**Table 3:** Percentage of positive quadrants on BOOST and LGE images for right and left pulmonary vein antrum (PVA)

Quadrants (%)	Right PVA BOOST	Right PVA LGE	Left PVA BOOST	Left PVA LGE
Superior	60	100	70	100
Antero-superior	100	100	90	100
Antero-inferior	60	100	60	100
Inferior	50	100	60	100
Postero-inferior	40	100	30	100
Postero-superior	60	100	70	100

### Quantitative lesion analysis

Regarding signal analysis, BOOST images exhibited higher values for SNR_,_ SBR, and CNR lesion/blood compared to LGE images. The CNR lesion/myocardium was in the same range between sequences: All data are reported in Table 4.

**Table 4:** Signal-to-noise ratio (SNR), signal-to-blood ratio (SBR), contrast-to-noise ratio (CNR) between lesion and blood (CNR lesion blood) and CNR between lesion and myocardium (CNR lesion myocardium) of acute atrial lesions for BOOST and LGE sequences

	BOOST sequence	LGE sequence	P value
SNR	19.8 ± 5.9	12.5 ± 5.3	0.02
SBR	2.9 (2.2–3.6)	1.7 (1.5–1.9)	0.02
CNR lesion blood	12.2 ± 4.5	5.3 ± 2.9	0.003
CNR lesion myocardium	4.6 ± 3.9	3.7 ± 2.3	0.5

*LGE* late gadolinium enhanced

Data are mean ± standard deviation or median (IQR)

The mean thickness of BOOST-positive quadrants was 3.8 ± 0.8 mm (range: 2.5–5.6 mm) on the right PV antrum and 3.9 ± 0.8 mm (range: 2.4–7.6 mm) on the left. These values were significantly lower than those observed in LGE-positive quadrants: 6.1 ± 1.3 mm (range: 3.5–8.3 mm) and 5.9 ± 1.8 mm (range: 2.9–10.1 mm) for the right and left PV antra, respectively (P < 0.001 for both). The mean thickness of microvascular obstruction (MVO) lesions was comparable to BOOST-positive quadrants on the right PV antrum (3.9 ± 1.3 mm, range: 1.6–7.0 mm; P = 0.5), but significantly lower on the left PV antrum (3.3 ± 1.0 mm, range: 1.3–7.4 mm; P = 0.02). The total volume of BOOST lesions (0.58 ± 0.29 cm^3^) was significantly smaller than that of both LGE lesions (11.57 ± 2.64 cm^3^; P < 0.001) and MVO lesions (1.88 ± 0.87 cm^3^; P = 0.01), as illustrated in Fig. 4. BOOST lesion volume showed a strong correlation with MVO lesion volume (r = 0.74; P = 0.015), but not with LGE lesion volume (r = 0.20; P = 0.8).

### Correlation with ablation parameters

**Fig. 5:**
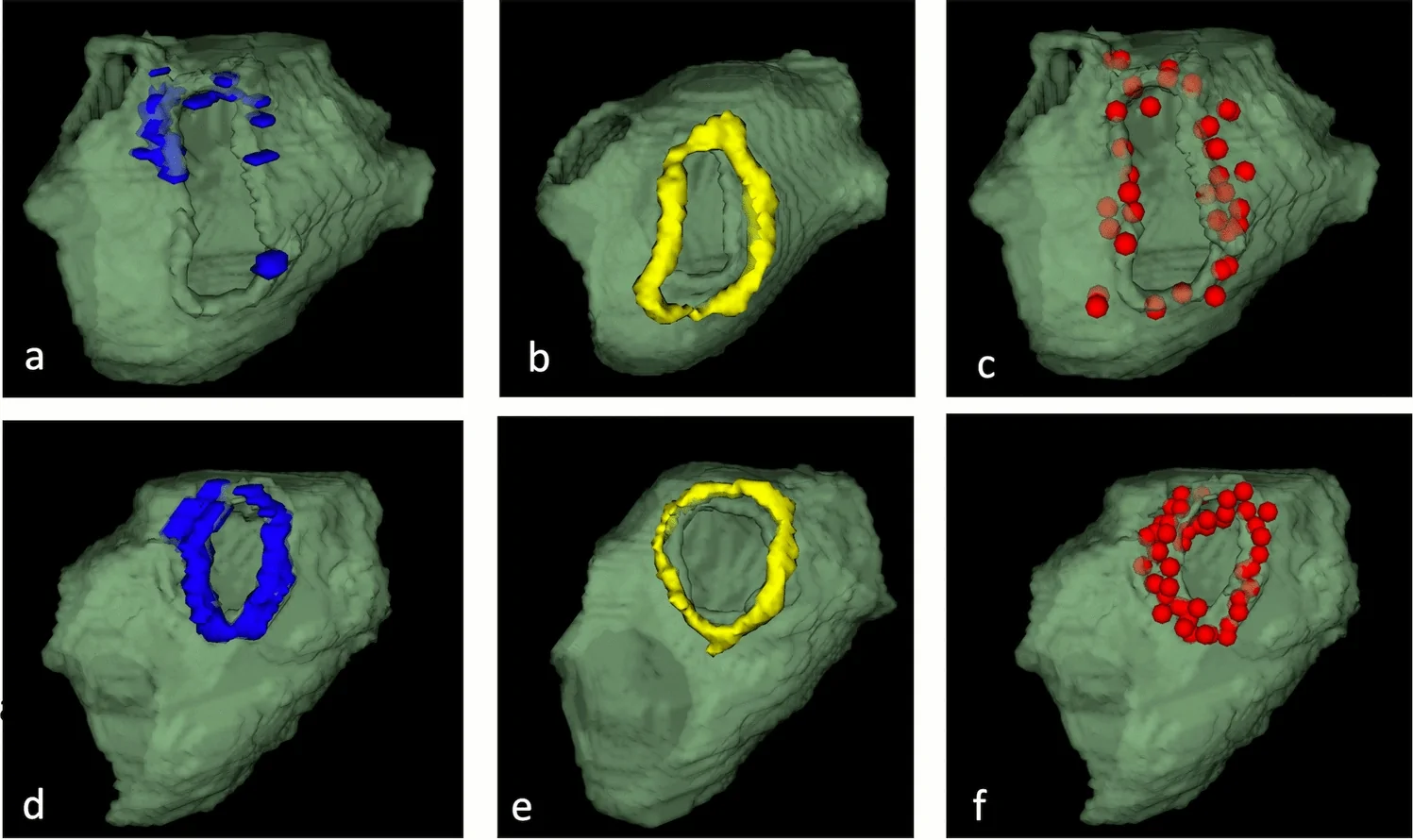
3D reconstructions of the left atrial antrum in two representative patients. Top row (patient 8): BOOST (**a**, blue), LGE (**b**, yellow), and ablation points (**c**, red) show discontinuous BOOST lesions, whereas LGE lesions appear continuous. This patient exhibited a wide inter-lesion gap (> 4 mm; mean across quadrants = 5.1 ± 1.3 mm) and a low impedance drop (< 10 Ω; mean = 5.2 ± 1.1 Ω), consistent with incomplete energy delivery. Bottom row (patient 7): BOOST (**d**) lesions appear almost continuous, and LGE (**e**) lesions appear continuous, both corresponding to closely spaced ablation points (gap < 4 mm; mean = 3.8 ± 0.3 mm) and a greater impedance drop (mean = 9.5 ± 2.6 Ω)

BOOST lesion continuity was associated with RFA procedural metrics (Fig. 5). Quadrants without or with discontinuous BOOST signal had significantly greater inter-lesion distance and a lower impedance drop compared to quadrants with continuous lesions (Table 5). The diagnostic performance of BOOST imaging for identifying ablation gaps > 4.0 mm yielded a sensitivity of 80%, specificity of 43%, positive predictive value (PPV) of 82%, and negative predictive value (NPV) of 40%. Similarly, for detecting an impedance drop < 10 ohms, BOOST demonstrated a sensitivity of 82%, specificity of 42%, PPV of 77%, and NPV of 50%.

**Table 5:** Correlation of cardiac MR findings with ablation parameters by pulmonary vein quadrant for BOOST and LGE images

	Continuous MVO on LGE (N = 80)	Discontinuous or absent MVO on LGE (N = 40)	P value	Continuous hyperintensity on BOOST (N = 30)	Discontinuous or absent hyperintensity on BOOST (N = 90)	P value
Maximum interlesion distance (mm)	4.4 (4.1–5.0)	4.5 (4.1–5.1)	0.73	4.2 (3.8–4.4)	4.5 (4.1–5.1)	0.007
Gap > 4.0 mm (n, %)	60 (75.0%)	32 (80.0%)	0.54	18 (60.0%)	74 (82.2%)	0.013
Minimum impedance drop (Ohm)	8.4 ± 2.7	8.4 ± 2.6	1.0	9.8 ± 2.5	7.9 ± 2.5	0.0006
Impedance drop < 10 Ohm	56 (70.0%)	28 (70.0%)	1.0	15 (50.0%)	69 (76.7%)	0.006

*LGE* late gadolinium enhanced, *MVO* microvascular obstruction

Data are mean ± SD, median (IQR) or n (%)

No significant correlation was found between RFA parameters and atrial wall thickness or lesion volumes on BOOST and LGE sequences.

### Reproducibility

For the detection of acute lesions within PV antrum quadrants on BOOST images, the agreement levels were moderate for both right (kappa: 0.60) and left (kappa: 0.50) PV antra. For lesion volume measurements, the ICCs between readers were moderate for BOOST measurements (ICC: 0.730 [95% confidence interval (CI), 0.356–0.942]) and good (ICC: 0.879 [95% CI, 0.514–0.970]) for LGE measurements.

## Discussion

All patients included in this study demonstrated high signal intensity lesions in the pulmonary vein antra on BOOST images, supporting the ability of the 3D T1-weighted BOOST sequence to detect acute lesions immediately after RFA at 3 Tesla, without the need for gadolinium administration.

This signal elevation is likely attributable to the paramagnetic effects of heat-induced methemoglobin and metmyoglobin, known to shorten T1 relaxation times in the early post-ablation period, as previously reported in animal models using unenhanced 3D T1-weighted sequences [20–22]. These changes may be more specific for acute tissue necrosis than conventional LGE sequences, which can reflect both necrosis and surrounding edema. Our results support this hypothesis: BOOST lesion volumes were significantly smaller than those measured with LGE and did not correlate with total LGE lesion volume, suggesting that myocardial edema detected by LGE is not captured by BOOST. Additionally, the observed correlation between BOOST lesion volume and microvascular obstruction (MVO) further supports this interpretation, as MVO has been shown to more reliably predict permanent scarring than conventional LGE shortly after ablation [16].

At the quadrant level, BOOST demonstrated lower sensitivity for lesion detection (~ 60%) compared to LGE, which achieved 100%. This limited sensitivity currently restricts the clinical applicability of BOOST as a standalone tool for comprehensive lesion mapping. To date, only one previous study has assessed unenhanced MRI for detecting acute atrial lesions immediately after RFA, using a long-TI-GRE inversion-recovery T1-weighted sequence, in animals [21] and in 10 patients [23]. This so-called TWILITE sequence achieved high detection rates (> 90%) in humans. It provides strong native T1 contrast but may also display a minor hyperintense component from surrounding edema, due to residual T2* weighting and the mixed T1/T2 dependence of the inversion-recovery GRE readout [20, 21]. In contrast, BOOST, being a GRE-Dixon-based black-blood T1-weighted sequence, primarily reflects T1 shortening related to necrosis and hemorrhagic by-products, and is less influenced by the edematous component. Finally, direct comparisons between BOOST and TWILITE sequences are limited by the lack of animal study with BOOST usually including optimal experimental conditions more favorable for lesion detection (higher energy delivery), and differences in magnetic field strength (3T in our study versus 1.5T for TWILITE in patients). In our study, 3T cardiac MRI was selected for its advantages over 1.5T in GRE/FLASH-based T1-weighted imaging, including improved signal-to-noise ratio and shorter in-phase and opposed-phase echo times for Dixon readouts [28, 29].

Despite its lower sensitivity for lesion detection compared with LGE, BOOST showed promising associations with procedural ablation metrics. Quadrants with absent or discontinuous BOOST lesions exhibited a significantly higher inter-lesion distance and a lower impedance drop, whereas such correlations were not observed with LGE. In our cohort, BOOST achieved a sensitivity of approximately 80% for identifying quadrants with a gap > 4 mm or an impedance drop < 10 Ohms, although specificity remained modest (< 45%). Because impedance drop reflects effective tissue heating and necrosis, and excessive inter-lesion distance (> 4 mm) is known to favor conduction gaps and pulmonary vein reconnection, these findings suggest that BOOST may better reflect areas of effective and durable lesion formation. In contrast, LGE likely captures a broader spectrum of tissue changes, including edema and transient injury, resulting in larger but less specific lesion volumes.

Several limitations merit consideration. First, the small sample size of this pilot study limits generalizability. Second, we did not assess BOOST signal persistence over time to distinguish transient versus stable lesions, as recently explored by van Pouderoijen et al. [30]. Third, pre-ablation BOOST imaging was not performed, precluding assessment of whether some high-signal areas may have been present prior to RFA. Finally, technical variability in acquisition and respiratory motion could influence image quality and lesion detectability.

## Conclusion

This proof-of-concept study illustrates the potential of native T1-weighted MRI for immediate lesion visualization. Our results showed that 3D T1-weighted BOOST imaging at 3T enables the detection of acute atrial lesions following RFA without the need for gadolinium contrast. Although BOOST did not sufficiently reproduce LGE for lesion mapping, its correlation with key RFA parameters and ability to capture more specific necrotic changes are promising incremental features obtained without contrast injection. Further validation in larger cohorts is warranted to clarify the diagnostic potential of these features and their potential role in guiding ablation strategies. With further optimization and integration into hybrid electrophysiology-MRI workflows, BOOST imaging could become a valuable adjunct for real-time procedural guidance in catheter ablation.

## Data Availability

No datasets were generated or analysed during the current study.

## Abbreviations

AF: Atrial fibrillation
BOOST: Bright-blood and black-blOOd phase SensiTive
CNR: Contrast-to-noise ratio
GRE: Gradient Echo
HB: Heartbeat
IR: Inversion recovery
LGE: Late Gadolinium Enhanced
MVO: Microvascular obstruction
PV: Pulmonary vein
RFA: Radiofrequency ablation
SNR: Signal-to-noise ratio
SBR: Signal-to-blood ratio
